# Influence of the Properties of Different Types of Recycled Aggregate on the Service Properties and Leaching of Paving Blocks Manufactured at Industrial Scale

**DOI:** 10.3390/ma17122898

**Published:** 2024-06-13

**Authors:** Miriam Hernández, Isidro Sánchez, Rosa Navarro, Marina Sánchez, Carlos Rodríguez

**Affiliations:** 1 Department of Sustainable Construction, Centro Tecnológico de la Construcción, Polígono Estrella, 30500 Molina de Segura, Spain; mirhernandez@ctcon-rm.com (M.H.); msanchez@ctcon-rm.com (M.S.); crodriguez@ctcon-rm.com (C.R.); 2 Civil Engineering Department, University of Alicante, 03080 Alicante, Spain; rosa.navarro@ua.es

**Keywords:** recycled aggregate, non-structural concrete, ionic leaching, principal component analysis (PCA)

## Abstract

The literature shows that a circular economy can benefit some sectors such as the construction industry. This sector demands huge amounts of raw materials and produces waste when buildings and structures are demolished. This paper explores the possibility of manufacturing at industrial scale paving blocks using different types of construction and demolition wastes as aggregates, without modifying the commonly used industrial conditions. A total of four different recycled aggregates were used in this research. Both natural and recycled aggregates have been characterized. The dosages were optimized (three different formulations). Prefabricated tests have been carried out on the products manufactured in industrial plants and the evolution of mechanical properties over time has been analysed. The results obtained were analysed statistically by applying the principal component analysis (PCA) method. To ensure the security of the elements manufactured, the ionic leaching of the materials used as recycled aggregate and of the elements produced has been tested. The main implications of this research on the construction industry show that the majority of recycled aggregates used could replace 25% of the natural aggregate in manufactured precast concrete, that the properties of the aggregates should be taken into account in the different standards and that all paving blocks manufactured in this study can be considered environmentally safe (no risk of leaching) according to the Netherland Soil Quality Decree. Therefore, it is evident that it is possible to manufacture on an industrial scale paving blocks with mixed recycled aggregates, concrete and ceramic in nature, both with the fine and coarse fractions that meet the requirements of its reference standard UNE-EN 1338 and the Netherland Soil Quality Decree that evaluates environmental risks due to leaching.

## 1. Introduction

The construction industry is one of the most natural resources consuming activities and one of the most polluting industries. As with any industry in the 21st century, there are many efforts to make it sustainable and to promote the circular economy [1,2,3,4,5,6,7]. Most of the efforts are focused on reusing industrial by-products of waste from different industries. Recent works have explored the inclusion of different types of inorganic industrial solid wastes, such as different types of slags, coal fly ash, red muds, tailings from the mining industry, etc. [8], even though most of these products have been incorporated for a long time in cement manufacture [9,10,11,12]. Some other papers incorporate waste as fibre reinforcements [13], including non-recyclable polymers [14]. In this paper, an industrial symbiosis indicator has been proposed to quantify the reuse of materials to promote the circular economy. However, most of the papers dealing with recycled elements in concrete are focused on the incorporation of different wastes replacing aggregates in concrete [15]. This could be a solution for cases where the production of natural aggregates is not possible [16]. Most of the recycled elements are construction and demolition waste (C&DW) due to the similar nature to natural aggregates or concrete, and the increasing production of this waste. In 2015, a paper was published in which it was shown that 25–30% of total waste generated in Spain was C&DW [17], so it seems essential to include this kind of waste as a new resource in the circular economy. The management of the C&DW is one of the key topics in the promotion of circular construction [2]. There are several applications of this waste as a filler for different uses [18], but these applications add little value to the resource. Other authors have used the C&DW for the incorporation of additions to the binder as fillers [19], or even recycling cement paste [20]. The most common way of recycling C&DW is by using it as recycled aggregate because it can be used in huge quantities and can be used to prepare concrete [21,22]. This is a high-added-value material that is also very widely used around the world. The use of recycled aggregates lowers the impact of concrete production on the environment [23]. The use of recycled aggregates is accepted by most European codes [24,25], but still finds difficulties in some other countries [26]. Prior to the use of the C&DW, it was necessary to classify the aggregates. The different constituents are measured using the standard UNE-EN 12620 [27], and the aggregates are classified as concrete, asphalt, and ceramic for aggregates where about 95% of the recycled aggregate has these constituents, or recycled mixed aggregate (RMA). RMA contains different percentages of the previous constituents and some others, like glass, plastic, etc., in small percentages. This type of recycled aggregate needs a less exhaustive classification process, which makes it easier to use this type of waste as a new raw material in the industrial production of concrete.

Most of the standards only focus on the type of recycled aggregate that can be used, but not much attention is paid to its properties. Thus, the Spanish standard [25] limits the use of recycled aggregate to coarse concrete recycled aggregate, and only the resistance to fragmentation (≤40) and the water absorption (≤5%) are limited in the standard. The use of other types of recycled aggregates is allowed in non-structural elements.

When recycled aggregates are used in concrete production, it is a well-known fact that the mechanical properties of concrete worsen, mainly due to the increase in the porosity of the concrete elements [28,29,30,31,32], or possibly to the existence of weak planes in the interface recycled aggregate/mortar [33]. It is also known that for non-structural elements, the replacement of a certain volume of natural aggregates by recycled aggregates can obtain the properties established in the standards for the different types of non-structural elements, and they could be used without any risk. For example, in [28,30,31,34], kerbstones, paving blocks and terrazzo tiles were produced to fulfil the requirements of the different standards. Most of the elements were produced using the vibro-pressing technique, which is the commonly used technique for these elements, including the incorporation of recycled aggregates of different types in the prefabrication industry [34]. It has to be pointed out here that most of the studies are performed under laboratory conditions, a previous step necessary for the industrial manufacture of elements. However, the industrial consumption of C&DW as recycled aggregates is the required step for circular construction. In this work, as will be stated in the experimental section, all the elements were produced in an industry without any change to the industrial manufacturing conditions.

It has been observed that there are discrepancies in the results observed by different authors using the same type of recycled aggregate, but very often attention has not been paid to the properties of the recycled aggregates, or at least not from a statistical point of view. One of the main techniques for the statistical analysis of experimental data is principal component analysis (PCA), which gives the combinations of the different experimental parameters that are relevant to the behaviour of the experimental data. This technique has been widely described [35], but it has not been much used in the recycled aggregate field. A recent paper uses PCA to explain the variability of the data in concrete that includes plastic aggregate in high-temperature environments, and the model proposed explains 70% of the variability and is considered excellent by the authors for predicting concrete strength [36]. In another paper, recycled concrete aggregate was used to produce concrete columns, named “recycled aggregate concrete filled steel tubular”. The variables analysed were different geometrical aspects of the column, the mechanical properties of steel tube, the percentage of recycled concrete aggregate and the compressive strength of concrete. In this case, the PCA was used to predict the compressive strength of different columns with good results. Another paper has studied the variability of the properties of recycled mixed aggregate on the mechanical properties of mortars prepared at a laboratory scale, using statistical methods [37]. In that paper, it appeared that there were no significative differences in the properties of the RMA collected during one year, and different methodologies were used to improve the behaviour of the mortars including RMA in their dosage. Some other applications have been found in the use of this technique as the use of PCA in the study of the mortar attached to the recycled aggregates to study the quality of the recycled material [38]. A search on Scopus using recycled aggregate and principal component analysis only yielded 16 papers, but none studied the influence of the properties of different types of aggregates on the properties of concrete. Most similar papers try to analyse the effect of the properties of recycled mixed aggregate (but just one type, with the same properties) on the properties of mortars, as was described before [37], and the second one tries to predict the behaviour when using 100% of the recycled aggregate for reinforced concrete columns, but no property of the recycled aggregate used was used as an independent variable [39]. PCA is used in this paper to study the influence of the properties of different types of aggregates in both the coarse and fine fractions on the properties of concrete blocks manufactured at an industrial scale.

In addition to the requirements established in the corresponding standard, the use of any type of waste in the construction sector must fulfil the environmental requirements established for the different concrete elements [40]. The incorporation of recycled aggregates in the production of concrete and precast concrete might increase the quantity of leached polluting elements [41,42,43]. This fact could cause an environmental risk due to the continued exposition of the polluting product to the surroundings [41,44]. For example, the presence of sulphur ions could cause the pollution of superficial and/or ground water [42], with a risk to the environment and the life in the surroundings. Gypsum, which is calcium sulphate, is commonly included in the C&DW, and it could leach sulphate ions into the environment [44]. For that reason, it is so important to analyse the leaching behaviour of the precast elements manufactured using recycled aggregates. The natural aggregates tend to show also ionic leaching when they are used for low-added-value applications.

Paving blocks usually have a ratio of external surface/volume higher than most of the concrete elements commonly used (foundations, continuous pavements, etc.). This high surface of the contact block/environment might cause an important leaching of the ions included in the concrete element. For example, in a recent work [45], the authors used rubber recycled from waste tyres, and in that paper, several solutions were proposed for the problem of leaching when those wastes were included as aggregates in asphalt mixtures. Leaching in concrete manufactured using recycled aggregates from C&DW has not been widely studied [41,42,44]. A search in Scopus for the words recycled aggregate, leaching and industrial gives only 17 results and in none were the samples prepared under industrial conditions, as in this work. Some works use recycled elements for road construction, but the aggregates are not encapsulated in concrete elements [46,47], while others study the leaching in concrete samples, but not ones that were vibro-pressed and manufactured under laboratory conditions [48]. However, the surface is smaller as compared to the recycled aggregate without encapsulating it, which may cause environmental problems due to leaching when the recycled materials are used in a lower-value application such as sub-base materials for road construction [18,49].

The objective of the paper is to study the possibility of using the fine and coarse fractions of RMA for the industrial-scale production of vibro-compacted paving blocks. To that end, different percentages of natural aggregate have been substituted by the same volume of several types of recycled aggregate under industrial conditions. The influence of the properties of the aggregates on the technological properties of the paving blocks has been studied using principal component analysis. Leaching behaviour has also been evaluated in recycled aggregates with no binder and paving blocks ready for use.

## 2. Materials and Methods

### 2.1. Materials

**Figure 1:**
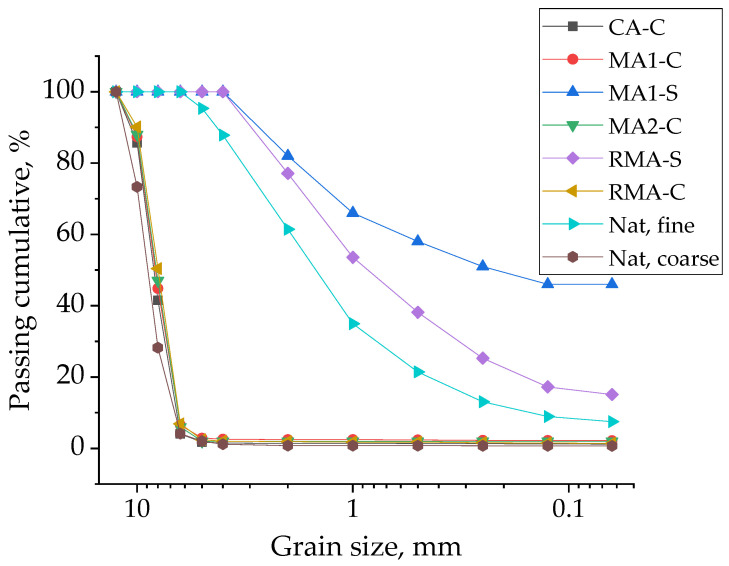
Granulometric distribution for each type of recycled and natural aggregate used in the study.

Samples were prepared under real industry conditions using the equipment and the workers of the company. The cement used was a commercial CEM I 42.5 R-SR according to the standard UNE-EN 197-1 [50]. The aggregates used were a natural aggregate and four different types of recycled aggregate. The recycled aggregates came from construction and demolition wastes and were provided by “Áridos y Prefabricados Barinas SL”, Abanilla (Spain). The fractions of aggregates used for the manufacture of paving blocks were fine (0–4 mm) and coarse (5–12 mm). They were classified as concrete recycled aggregate (CA) (coarse fraction), two different types of masonry recycled aggregate (MA1 and MA2) (one of them, MA1, in the coarse and fine fractions), and a recycled mixed aggregate (RMA), also in the fine and coarse fractions. The natural aggregates were used also in both fractions and were provided by a quarry in Lorca, Murcia (Spain). The nomenclature used and the main properties of the aggregates used are shown in Table 1. The standard used for the measurement of each property is also included in the table. The particle size distribution of coarse and fine fractions is shown in Figure 1.

The composition of each type of recycled aggregate is shown in Table 2.

**Figure 2:**
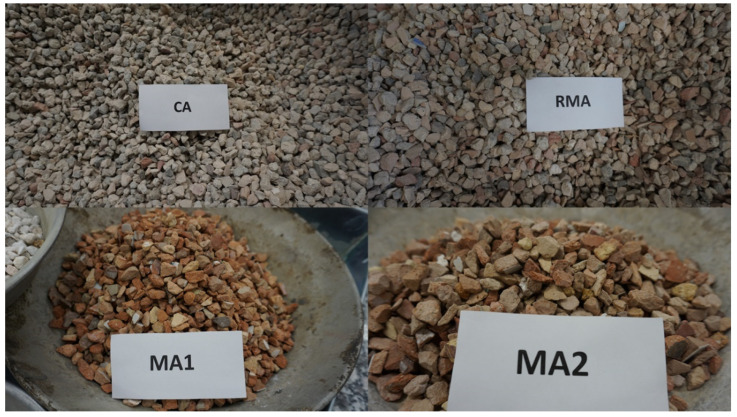
Recycled aggregates used.

As could be expected, after the classification of the C&DW at the plant, both types of masonry aggregates were 100% ceramic material (bricks mainly). The CA contained mainly concrete and unbound aggregates, with a low value of fine floating particles. The RMA includes every type of admissible C&DW, including asphalt. A picture of every type of recycled aggregate is shown in Figure 2.

### 2.2. Dossage under Industrial Conditions

The dosage was optimized to be used with no change in the industrial conditions of the manufacture of the paving blocks. The natural aggregates were replaced by each of the different types and fractions of recycled aggregate studied to obtain the maximum volume fraction of recycled aggregate that can be used without any significant loss of property. The percentages of recycled aggregate were chosen as 25, 60 and 100% of the volume of natural aggregates. A set of blocks with 100% natural aggregates was also prepared to obtain the reference values.

The dimensions of the paving blocks are 20 × 10 × 6 cm^3^, and the fabrication process consists of the production of two layers. The first one has a thickness of 5.5 cm, while the second one is only 0.5 cm thick. Recycled aggregates have only been included in the base layer, with 5.5 cm, because is the one with higher aggregate consumption, and is the one that mainly includes coarse aggregates.

The dosage of the different paving blocks prepared is shown in Table 3. The nomenclature includes the type of recycled aggregate used (CA for concrete, MA for masonry, RMA for mixed), the fraction of recycled aggregate used (C for coarse, or S for fine), and the percentage of recycled aggregate used. Control is used for the samples with 100% natural aggregates.

Keeping in mind the idea of the industrial use of recycled aggregates, the aggregates were not saturated before use, as is common in laboratory studies [55], but not under industrial conditions. The total water for each type of recycled aggregate was adjusted taking into account the water absorption of each aggregate type to ensure the same amount of efficient water for cement hydration.

The blocks were vibro-pressed. Vibro-pressing has already been described [30,56] and is a process that combines pressure and vibration to cast the concrete in metallic moulds. This technique can only be used with dry consistency concretes, so the slump cone was determined for every mix according to the standard UNE-EN 12350-2:2020 [57].

**Figure 3:**
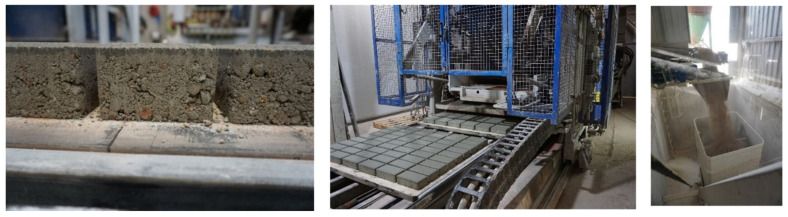
Manufacture of paving blocks, under industrial conditions.

A picture of the industrial manufacturing of paving blocks can be seen in Figure 3. Once manufactured, the blocks were kept in the curing area of the company until testing.

### 2.3. Specific Tests for Paving Blocks

As was stated before, all the specific tests required for the commercial distribution of the paving blocks were performed.

The density was determined at 28 days using the standard UNE-EN 12390-7 [58].

According to the standard UNE-EN 1338 [59], several properties of the paving blocks were determined: the mechanical resistance at the ages of 28 and 90 days, the water absorption of the blocks at 90 days, and resistance to abrasion and slipping at 90 days (on the side containing recycled aggregates). In total, eight samples were tested for each property and each curing age.

### 2.4. Statistical Analysis

As will be shown later, the results of the same type of recycled aggregate, either on the coarse or the fine fraction, were different for the industrially produced blocks. To understand the relevance of the different properties of the aggregates on the final result of the manufactured products, principal component analysis (PCA) has been conducted on the mechanical properties, water absorption, density and abrasive resistance of the manufactured elements. PCA was initially used at the beginning of the 20th century [38]. One of the main uses is to transform a set of inter-related variables into a reduced number of independent variables (principal component, or dimension) that are linear combinations of the original variables [60]. Each one of the dimensions includes a certain percentage of the total information included in the set of inter-related variables. Each principal component is associated with a percentage of information included in the complete data set, and the different relevance of each variable can be represented in a correlation circle that represents the inter-influence among the variables and their relative weights. The PCA also implies a reduction in the number of variables. As was said in the introduction, Pawar et al. [36] established 70% as the minimum percentage of the PCA to have enough information to predict the behaviour of concrete samples, so when it is possible, only two dimensions will be taken. All the calculations and the graphical representations have been conducted using RStudio and the different R libraries. The authors assume that the number of samples established in the standard [59] will be statistically representative. To check the validity, and being conscious that it is absolutely impossible to manufacture new samples, the authors have prepared a set of 30 new random results that fulfil the average value and the standard deviation of the experimental results. PCA has been conducted on the experimental data (8 samples) and the extended set of data, including the experimental and the simulated results (38 samples) and no significative difference has been found in the PCA of both sets. The comparison has been conducted for two different properties (mechanical strength and water absorption of the blocks) where the coarse (mechanical properties) or fine fraction (absorption) were replaced.

### 2.5. Ionic Leaching

The use of waste is dangerous from an environmental point of view, but the toxicity may decrease when the C&DW is introduced in the paving blocks. To check this hypothesis, both the leaching behaviour of recycled aggregates and blocks including 100% recycled aggregate were tested according to the standard UNE-EN 15863 [61]. The test procedure determines the release of the inorganic components of a monolithic waste as a function of time when it comes into contact with an aqueous solution (distilled water) by simulating a long-term scenario. The test specimens, paving blocks of 20 × 10 × 6 cm^3^, were completely immersed in closed plastic containers with distilled water so that the tops of the samples were submerged by at least 2 cm and the distances between the test portions and the vessel walls were at least 2 cm. The lixiviant was introduced into the vessel and renewed at predetermined time intervals as established in the standard. The eluate was collected in 8 separate fractions, measured for pH and conductivity, filtered, and, using plasma mass spectrometry and chromatography, the components specified by the European Landfill Directive were quantified [40]. The test procedure was repeated with the recycled aggregates before being used for block manufacturing to test the retention capacity of the manufactured concretes against leaching.

## 3. Results

### 3.1. Mechanical Resistance

**Figure 4:**
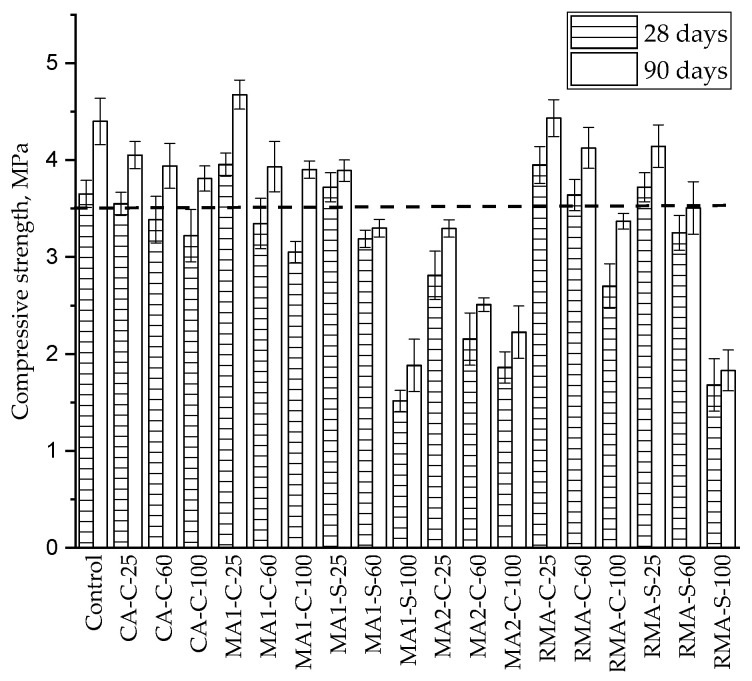
Results of mechanical strength in paving block after 28 and 90 days for every type and percentage of recycled aggregate.

According to the standard UNE-EN 1338, annex F [59], the mechanical resistance was determined in eight blocks for age and type. The results of the average resistance and the standard deviation are shown in Figure 4 for both 28 and 90 days. In this figure, a line also appears for the minimum 3.5 MPa, established by the standard as the minimum value admissible.

The analysis of the results will be conducted first for the use of recycled aggregate as coarse aggregate in the precast concrete, and later the influence of using fine aggregates will also be analysed.

#### 3.1.1. Coarse Aggregate

The highest resistance is given by sample MA1-C-25. In these samples, 25% of the coarse natural aggregate had been replaced by the same volume of masonry coarse recycled aggregate. The mechanical strength of these samples is 6.2% higher than the strength of the control samples. On the other hand, the compressive strength of the specimen prepared with 25% masonry aggregate MA2 shows a lower value, even below the minimum value established by the standard.

To try to understand the influence of the properties of the recycled aggregate on the resistance of the paving blocks, principal component analysis (PCA) has been conducted on samples where the coarse aggregate has been replaced, as well as on those where the replacement took place in the fine fraction.

#### 3.1.2. PCA of Samples with Coarse Recycled Aggregate

To perform PCA, the variables considered were the following: density, resistance to fragmentation, water absorption, content in floating particles in the aggregates, percentage of substitution in the coarse fraction, and resistance of each block. The values for these variables have already been previously shown in Table 1 and Figure 4. The study will be applied to both the results obtained at 28 days and at 90 days. In the specimens cured for 28 and 90 days, the PCA shows the results shown in Table 4.

**Figure 5:**
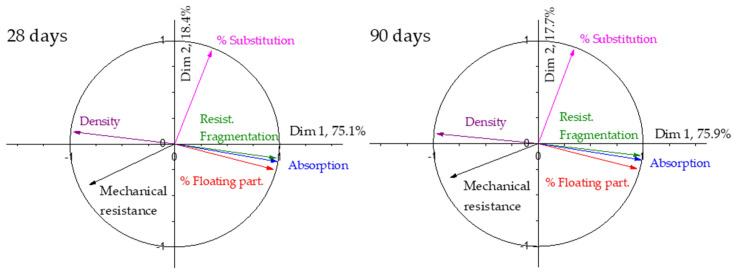
Correlation circle for the samples with replacement of coarse aggregate at 28 and 90 days.

Since a total of six variables have been considered, the sample space has six dimensions. The eigenvalues are the relative weights of each eigenvector or linear combination of variables that form the principal components. The variance is the percentage of the results that can be explained using the linear combination of variables included in the given dimension. As can be seen in Table 4, the first dimension explains more than 75% of the experimental data, more than enough for making predictions according to [36]. With the first two dimensions, the cumulative variance increases to more than 93%, a very high value, and it implies that most of the results could be explained with these two dimensions. The correlation circles for this data set are shown in Figure 5.

As can be seen in Figure 5, the first dimension is mainly composed of the aggregate properties and the mechanical properties of the blocks. The density and the mechanical strength show similar tendencies and are opposite to the rest of the properties. It means that, as could be expected, an increase in the density of the aggregates increases the mechanical strength of the blocks, and an increase in the resistance to fragmentation, water absorption and percentage of floating particles in the aggregate makes the mechanical strength decrease. The second dimension (which explains over 18% of the variations in the results at both 28 and 90 days) is mainly represented by the percentage of substitution in the coarse fraction. The correlation circle can be obtained from the contribution of each property to each dimension, drawing dimension 1 vs. dimension 2. As an example, the data for 28 days in Figure 5 are shown in Table 5. In this case, the data are presented to show equivalence and the correlation circle will be used from now on. Although only dimensions 1 and 2 appear in the circle, Table 5 includes the contributions of each property to the rest of the dimensions. The values of dimension 6 are so insignificant that the program does not give them. Based on the values obtained, it is confirmed that the most important contributions are the first two dimensions.

The water absorption has a slightly higher relevance in the first dimension, and the rest of the aggregate properties have a weight higher than 0.94; in the second dimension, the main relevance is for the percentage of substitution, as could be inferred from the correlation circle. Thus, according to the results obtained, the properties of the recycled aggregates show a great influence on the results of the blocks manufactured with recycled aggregates, even higher than the degree of replacement.

As can be seen in Table 4, the relevance for each dimension is very similar at both 28 and 90 days, slightly higher than 90 days for dimension 1 and slightly lower for dimension 2. Between them, more than 93% of the results are explained. Figure 5 shows that both have very similar behaviour, and as expected, time influences the results, but the influence of the properties of the aggregates and the percentage of substitution are maintained over time. It is true that the weights of each property change, but very slightly and not perceptibly.

#### 3.1.3. Fine Aggregate

Regarding the influence of the recycled aggregate in the fine fraction, only the masonry type 1 (MA1-S) and the mixed C&DW (RMA-S) were used. As can be seen in Figure 4, using a 25% content in both recycled fine aggregates gives similar results to the control sample, and the compressive strength is higher than the minimum value required by the standard. The use of 60% recycled aggregate in the fine fraction gives a bad result from the mechanical point of view, and the blocks with 60% or more of fine recycled aggregate could not be used, attending to the standard. Compared with the control sample, the use of 100% recycled fine aggregate causes a decrease of 58.4 and 54.0% for masonry and mixed aggregates, respectively, after 28 days and of 57.2 and 58.4 after 90 days.

#### 3.1.4. PCA of Samples with Fine Recycled Aggregate

The same principal component analysis has been carried out on samples with fine recycled aggregate. The results for the eigenvalues and the percentage of variance in the results explained by each dimension are shown in Table 6 for 28 and 90 days.

In the case of using fine aggregates, the variance represented by the first dimension is lower than in the case of using coarse recycled aggregates, but it is still close to 70% at both curing ages. The second dimension has greater relevance in the variance of the results and explains 23.7% and 21.7% of the results at 28 and 90 days, respectively. As occurred with the coarse aggregate, the first two dimensions explain more than 90% of the results, so they can explain the variance of the result.

**Figure 6:**
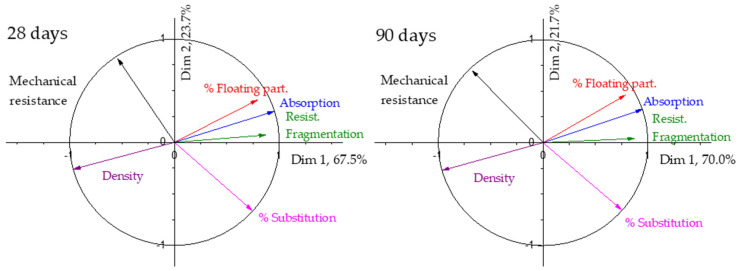
Correlation circle for samples with fine recycled aggregate at 28 and 90 days.

The correlation circle for the samples with recycled fine aggregate at 28 and 90 days is shown in Figure 6. As can be seen, the mechanical strength is more influenced by the degree of substitution, which has a higher contribution to dimension 1, as is clearly seen in the correlation circle.

As can be seen, there are no differences in the analysis of the results of the fine recycled aggregate from 28 to 90 days. The properties of the aggregate continue to have a decisive relevance on the properties of the manufactured concrete blocks, but less than in the case of recycled coarse aggregate. The degree of substitution has a greater importance when fine recycled aggregate is used.

### 3.2. Water Absorption

**Figure 7:**
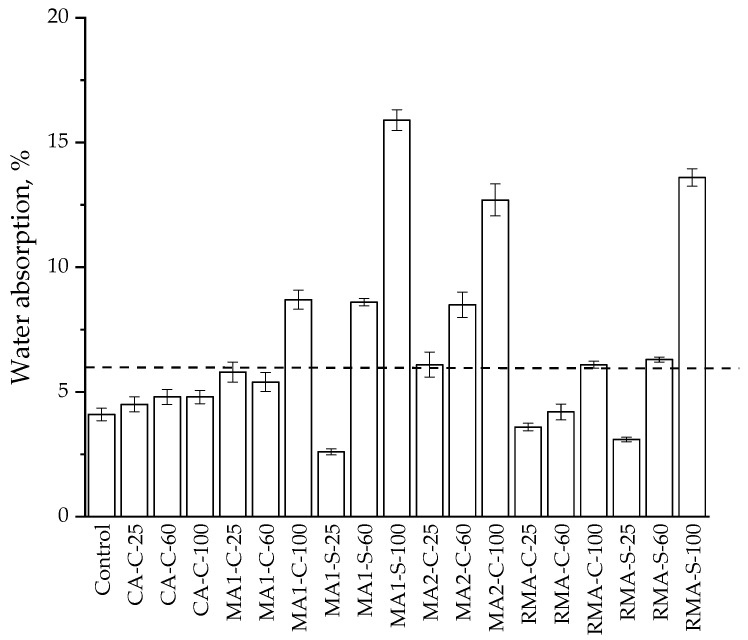
Results of water absorption of paving blocks for every type and percentage of aggregate used.

Due to the mechanical behaviour shown in the samples, previously described, the water absorption tests were only carried out on samples after 90 days, since they had compressive strength values above the minimum value required by the standard. The results of the water absorption are shown in Figure 7. In addition, this figure shows the average value and the standard deviation obtained from the eight determinations. The standard UNE-EN 1338 [59] states a maximum value of 6% to be tagged as B or class 2. This maximum value has also been included in Figure 7.

A higher absorption of water could be expected for every block manufactured with recycled aggregate due to the higher porosity of the C&DW compared with the natural aggregate (see Table 1). The first result of relevance is that excepting the paving blocks prepared using masonry recycled aggregate MA2, every sample prepared with 25% of recycled aggregate could be tagged as B (class 2) according to the standard UNE-EN 1338 [59], a fact that means a good climatic resistance, which is a very important aspect for these kinds of blocks. This result is coincident with the mechanical strength results, and previous results [28], where kerbstones were prepared using RMA, and every sample with less than 50% of RMA could be tagged as B, as is the case in this work.

From the previous discussion it seems clear that the water absorption is also highly influenced by the aggregate properties and to study the influence of the different properties on the water absorption the principal component analysis has also been conducted on these data.

Table 7 shows the results for the dimensions and variance justified by any one of these dimensions in the case of the coarse and fine fractions. As can be seen, the results are very similar to those obtained for the mechanical strength, with almost 94% of the variance in the results explained by the first two dimensions with the coarse fraction. In the fine fraction, the contribution to the variance of the results of dimension 1 is smaller, and higher for the case of dimension 2.

**Figure 8:**
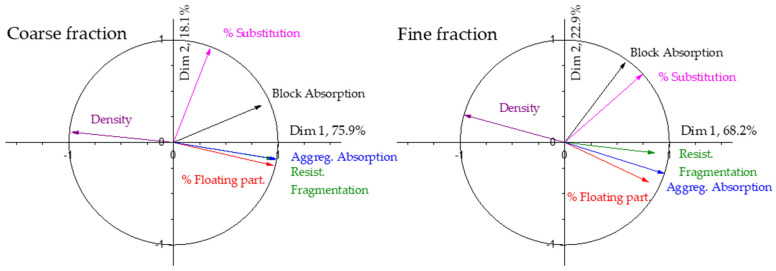
Correlation circle for the results of water absorption of samples manufactured using coarse and fine recycled aggregate at 90 days.

Figure 8 shows the correlation circle obtained from the results of absorption. As can be seen, the first dimension is mainly composed of the aggregate properties. The density of the aggregates shows a behaviour opposite to the block absorption, while the rest of the properties (floating particles, aggregate absorption and resistance to fragmentation) make the water absorption rise as they increase, as could be expected. The highest weights of the properties are for the aggregate absorption (0.98) and the aggregate density (−0.97) in the specific case of the coarse fraction. The degree of substitution is mainly responsible for dimension 2, and, as happened with the mechanical strength, there is a clear influence of the degree of substitution on the water absorption of the paving blocks, but lower than the influence exerted by the aggregate properties. If the results for the coarse fraction and the fine fraction are compared, it can be seen that the composition of each dimension is similar in both cases, but the block absorption is closer to the percentage of substitution in the fine fraction.

### 3.3. Density, Abrasive Resistance and Slipping Resistance

The properties of the different types of aggregates are summarized in Table 1. There, it can be clearly seen that recycled aggregates, of any type, show a lower density compared to natural aggregates. This result has already been reported in previous works [31,62]. All the results of density, abrasive resistance and slipping resistance of the manufactured paving blocks are summarized in Table 8.

As can be seen, the value of the density of the manufactured paving blocks is directly related to the density of the coarse aggregates used, either natural or recycled. Among the recycled aggregates, the one with the higher density is that coming from concrete waste (CA). Paving blocks prepared with this type of recycled aggregate show a density only slightly lower than the control blocks. This result is in agreement with the results of mechanical strength and water absorption, where the density of the aggregates played an important role. On the other hand, aggregates tagged as MA2 show the lowest density among all the aggregates and produce the paving blocks with the lowest density and the worst results in terms of water absorption and mechanical strength.

The data of abrasion resistance and slipping resistance determined at 90 days are also shown in Table 8. The values for the abrasion test are very low, a fact that could be due to that the test has been conducted on the base layer (5.5 cm thickness), which is the layer where the recycled aggregates were used. The abrasive resistance of the samples manufactured using recycled aggregates was very similar for any type of recycled aggregate, except in the case of using the ceramic recycled aggregate tagged as MA2-C.

The slipping resistance of paving blocks manufactured using recycled aggregate does not change much compared with the control blocks, and this result does not depend on the type of aggregate and, as a consequence, on the properties of the recycled aggregate used, or the percentage of recycled aggregate.

#### 3.3.1. PCA of the Block Density Measurements

Table 9 summarizes the dimensions given by the PCA applied to the density values obtained by the manufactured paving blocks using the coarse fraction. As can be seen, the first two dimensions only summarize 83% of the results, but, as was stated in [36], a value above 70% has a significant relevance.

**Figure 9:**
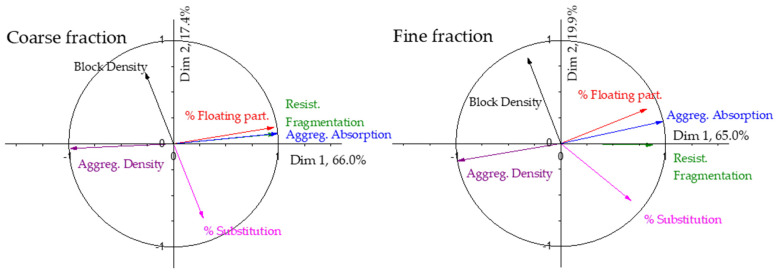
Correlation circle for the results of the density of the blocks manufactured in the study at 90 days. Influence of the coarse and fine aggregates.

The correlation circle is shown in Figure 9.

In Figure 9, the correlation circle shows the influence of the coarse aggregates on the density of the blocks. The influence of the aggregate parameters is less relevant (they are almost orthogonal to the block density arrow), and the main relevance comes from the substitution degree, which, as could be expected, shows a behaviour opposite to the block density (higher substitution, lower density).

When using recycled aggregates in the fine fraction, the results are very similar. The correlation circle is also shown in Figure 9. The results are very similar to the use of the coarse fraction, but the block density and the percentage of substitution are not so aligned, indicating not such a direct correlation, possibly due to the change between using 25 and 60% or 100% of fine recycled aggregates.

#### 3.3.2. PCA of the Abrasion Test

The data were analysed as with most of the data included in the paper, and Table 10 summarizes the dimension results of the PCA. As can be seen, with two dimensions, more than 90% of the results can be explained, with dimension 1 being the most relevant, as usually happens.

**Figure 10:**
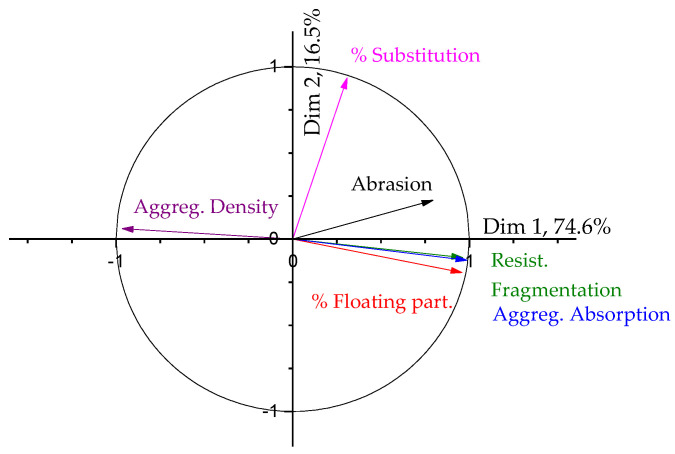
Correlation circle for the results of abrasive wear of the blocks manufactured in the study at 90 days with the coarse fraction.

The covariance circle is shown in Figure 10. As it can be clearly seen, dimension 1 is mainly composed of the properties of the aggregates and is the one with the highest influence on the abrasion resistance of the blocks, while dimension 2 is mainly integrated by the substitution degree and has a lower relevance on the abrasion resistance, as suggested by the initial analysis of the average data.

### 3.4. Leaching Test

The leaching study was determined both in the recycled aggregates used and in the paving blocks made. This comparison is very interesting because if the leaching of ions is reduced when recycled aggregates are included in monolithic blocks, it will mean that not only companies can use this type of waste, reducing the consumption of natural aggregates in terms of the required properties of the paving blocks, but also the environmental risk of pollution due to the stocking of construction and demolition wastes. To that aim, the lixiviation of the recycled aggregates of each type was measured, and the lixiviation of the industrially manufactured paving blocks was also tested after 90 days. The results show the possibility of using these paving blocks in a safe way for the environment and society.

#### 3.4.1. Recycled Aggregate

Table 11 shows the concentrations of metals, chloride and sulphate in the leachate and the classification according to the EU Landfill Directive [40] of the raw recycled aggregates used in this study. Most of them could be used for landfilling according to the mentioned EU directive, but landfilling should only be an option for wastes that cannot be introduced in the circular economy as a new resource.

The Landfill Directive classifies the wastes into three different groups of wastes (inert, non-hazardous and hazardous) according to their pollutant potential. Almost every type of recycled aggregate used in this study shows a value of leached metals lower than the ones required by the European standard to be classified as “inerts” [40]. Only the MA2 type of ceramic aggregate exceeds the chromium limit values for leaching. On the other hand, the ceramic sample MA1 does meet the limits for chromium. This difference could be due to the higher porosity and lower density of the MA2 aggregate compared to the MA1 aggregate, which promotes the leaching of its compounds.

On the other hand, the value of sulphate ions is higher than the limit for the classification as inert for every type of waste used, except for coarse aggregates coming from concrete, CA-C. The MA1 and MA2 aggregate types show sulphate concentrations five times higher than those required by the normative for being classified as “inert”. In the case of mixed recycled aggregates (RMA), the leaching value of sulphates varies depending on the fraction of aggregates used. RMA coarse aggregate has a sulphate leaching similar to that observed for ceramic aggregates, while the leaching of RMA fine aggregate is twice that of coarse aggregate. With these results, every C&DW could be classified at least as non-hazardous.

Similar results were observed by [43], where 17 different types of aggregates from C&DW were analysed. Every type of aggregate, except those from concrete, exceeded the sulphate limits to be considered inert. Regarding the metals, almost every aggregate in this study could be classified as inert, except for the aggregates of ceramic nature that exceed the leachate of chrome.

Regarding the metal content in the leachate, in another study [41], blocks were prepared using both the fine and the coarse fraction of recycled mixed aggregate formed by concrete, ceramic tiles, Arabic tiles, terrazzo, and small gypsum or wood pieces, and the leaching study showed that from an environmental point of view, the quantities of harmful elements-substances are within the limits stipulated in the German standard [63].

#### 3.4.2. Paving Blocks

The leaching of environmentally relevant compounds from concrete is mainly controlled by diffusion mechanisms [16,17,18]. So, to reduce the leaching of C&DW, one simple way could be to use them to produce concrete samples. In the case in which the leaching is reduced, two goals are reached: reducing the consumption of raw materials and reducing pollution, both by using wastes as materials to produce concrete and also by reducing the potential leaching of polluting substances into the environment.

pH plays an essential role in the leaching ability of chemicals. In a study, the behaviour of concrete with a pH from 7 to 4 was evaluated [18]. In that work, it was noticed the difference in behaviour was a function of the pH value. Two different groups of elements were identified. One group was formed by the elements that formed oxyanions (Mo, Se, As, Sb and Cr) and the second group was formed by elements that released metallic cations (Zn, Cu, Ni, Pb and Cd). The amount of metallic cations was higher at pH 4, while the oxyanions were leached in higher concentrations at pH 7. The pH level of the medium where aggregates are kept is important. In the case of this study, the blocks will show an alkaline pH, with a tendency to decrease with time to a value of 7–8 due to the natural carbonation process [19]. The blocks were submerged in pure water with a pH value of 7.

Table 12 shows the concentrations of metals, chloride and sulphate in the leachate of the paving blocks. Every single paving block manufactured with these recycled aggregates in real industry conditions can be considered non-hazardous to the environment according to the Netherland Soil Quality Decree (NSQD) [64]. The NSQD establishes the requirements that monolithic construction products and soils must meet to be used in construction in order to avoid the possible environmental risks produced by leaching.

## 4. Discussion

### 4.1. Mechanical Resistance

Regarding the analysis of the resistance using recycled aggregate, it has to be said that many standards or codes only allow the use of the C&DW as coarse aggregates. As could be expected, the value of breaking resistance decreases as the fraction of recycled aggregate increases, as has been widely reported [30,31,32,65]. The loss of mechanical properties is usually associated with the lower resistance of recycled aggregates compared to natural aggregates. However, after 90 days, all types of paving blocks that include 25% and 60% recycled aggregate, except the MA2 recycled aggregate, fulfil the resistance requirement established in the standard UNE-EN 1338 [59]. This result also includes samples with 25% of fine recycled aggregate.

Most of the standards [24,25] establish a classification for the recycled aggregates depending on the source, i.e., recycled concrete aggregate, recycled ceramic aggregate, recycled asphalt aggregates or mixed recycled aggregates, and establish different percentages of replacement for each type of waste used. In this case, it seems clear that the loss of mechanical resistance cannot be attributed to the nature of the recycled aggregate, as is included in the literature [66,67,68,69,70] and the standards [24,25]. In this research, two coarse aggregates that come from masonry waste, MA1 and MA2, have been used. As can be seen, there are important differences in the properties of these aggregates, included in Table 1. The density for the recycled aggregate MA1 is 2.15 g/cm^3^, while for MA2 it is only 1.85 g/cm^3^. The resistance to fragmentation (dimensionless property, related to the hardness of the aggregates) has a value of 30 for MA1 and 40 for MA2. So MA2 is an aggregate more brittle than MA1. Also, the water absorption, related to the porosity, is almost double for the recycled aggregate MA2 (17.3%) compared to the water absorption of aggregate MA1 (9.7%). So, the total amount of water used for samples manufactured replacing natural aggregate by masonry recycled aggregate MA2 include a higher amount of water. With this analysis, it can be stated that the final properties of concrete do not only have to do with the composition of the recycled aggregate. The physical and mechanical properties of the recycled aggregate should be taken into account to estimate the feasibility of using the waste as an accurate recycled aggregate.

In order to study the influence of the aggregate properties and the percentage of substitution in the final strength of the manufactured blocks, the PCA technique was used on the collected experimental data and the correlation circle is shown in Figure 5. As can be seen there, the vector of the mechanical resistance is mainly a composition of the density and the percentage of substitution. The lower the density of the recycled aggregates, the lower the resistance of the paving blocks, and the higher the substitution degree, the lower the mechanical resistance of the paving blocks. This could be the reason why some standards limit the maximum amount of recycled aggregates [24,25] and the result of the influence of the density has also been widely shown. The rest of the variables show an influence absolutely opposite to the influence of the density and resistance of the manufactured blocks. The results obtained at 90 days, as well as the PCA for those results, show no change, only the effect of time. This result is coincident with most of the papers published [28,71,72,73,74], except for those where a self-curing effect has been reported, due to the ability of the recycled aggregate to accumulate water and release it with time, improving in an important way the properties of concrete [30]. This fact does not happen in this work.

Regarding the results of the blocks that include fine aggregates, the use of 100% of recycled fine aggregate causes a decrease of 58.4 and 54.0% for masonry and mixed aggregates, respectively, after 28 days and of 57.2 and 58.4% after 90 days. For the same aggregate, but using the coarse fraction, the replacement of 100% of the aggregate shows only a mechanical loss of 16.4 and 26.2% at 28 days and of 11.3 and 23.4% at 90 days. This fact could be due to the difference in the granulometric distribution shown in Figure 1. As can be clearly seen in that figure, the fine recycled aggregate shows a high volume of particles with a diameter below 1 mm. For fine natural aggregates, only 35% of the particles have dimensions below 1 mm, while for masonry fine recycled aggregate, the percentage is 66% and for recycled mixed aggregates it is 54%. The presence of fine particles and the consequences such as water absorption of the recycled fine aggregates seem to be the main causes for the limit of 25% of replacement in the manufactured blocks. The use of recycled fine aggregate has been a point of divergence in the results of several researchers [29,75,76], but the results presented in this set of tests show that from the mechanical point of view, it is possible to replace 25% of the natural fine aggregate by any of the two types of recycled aggregates used at industrial scale.

Regarding the mechanical behaviour of blocks, similar results have been presented by other authors [31,77,78,79]. The main difference comes from the fact that most of the tested elements were produced under laboratory-controlled conditions, and in this paper, the elements have been produced in real industrial plants with real manufacturing conditions, which is an essential step for the massive recycling of C&DW.

No significant differences have been found among the results of 28 and 90 days. For every sample prepared, the mechanical behaviour slightly improved with curing time, as could be expected, especially after the PCA of the samples with coarse aggregates, but no self-curing was seen [80,81] because there is no significant gain of mechanical strength with time, as happened in [30].

The analysis of the influence of the use of recycled fine aggregate on the mechanical properties of industrially manufactured paving blocks is mainly influenced by the degree of substitution, and not so much by the density, as happened with the coarse aggregate. These results confirm the difference in behaviour among coarse and fine recycled aggregate widely reported by some authors [77,82,83].

So, as a general conclusion from the mechanical point of view, with the different types of recycled aggregates used it can be said that the properties of the aggregate have a great influence on the final mechanical resistance of the manufactured concrete blocks, and should be taken into account in the different standards. In total, 25% of the studied recycled aggregate types could be used as a replacement for natural aggregate, fulfilling the values of mechanical strength required by the standard for paving blocks.

### 4.2. Water Absorption

As can be seen in Figure 7, the samples with 25% of the fine recycled aggregates of each type and the samples with the mixed recycled aggregate replacing 25% of the coarse fraction present values of water absorption lower than the control sample. However, when the fraction of fine recycled aggregate increases, the value of the water absorption rises, and the samples with 100% of recycled fine aggregate exhibit the highest values for water absorption. If the results of the samples with a 100% fine fraction are compared with those with a 100% coarse fraction, it can be seen that the water absorption of the paving blocks manufactured with coarse recycled aggregate shows a much lower water absorption, especially in the case of using RMA that is close to the reference value of 6%. This result had already been reported with another type of recycle aggregates [31] used in different concrete elements, and it was established that the percentage of fines was a factor with a high influence on water absorption. The results of that work were promising, in contradiction with some previous results [29,84].

The PCA again shows the difference in behaviour among replacing coarse aggregates or fine aggregates in the blocks. While samples with coarse recycled aggregates show a high influence on the absorption of the recycled aggregates, in agreement with most of the published works in the field [28,74,85], the use of fine recycled aggregates shows a high influence of the percentage of substitution (see Figure 8). Block absorption and percentage of substitution appear almost aligned in the correlation circle, meaning a huge influence, while the rest of the properties are almost perpendicular to the block absorption vector, meaning that the block absorption is almost independent of those properties. This result has never been published, as was stated in the introduction, and more effort is required to understand the reason for this behaviour.

### 4.3. Density, Abrasive Resistance and Slipping Resistance

These results will be discussed by property.

Regarding the density, it is only noticeable that in the case of using the recycled aggregates in the fine fraction, the density of the blocks is mainly affected by the density of the recycled aggregates up to 60% replacement. For 100% replacement in the fine fraction, the value of density is much lower compared to the reference value. It had already been reported in a previous study [22], and this agrees with the relevance of the degree of substitution in the properties of the blocks already reported in the PCA of the data included in this paper.

Regarding the abrasive resistance, the use of 25% recycled aggregate causes an increase of about 1 mm in the wear resistance of the samples, while the use of 60 and 100% causes differences of about 8 and 12 mm. This result could be due to the influence of the substitution degree or just to some properties of the recycled aggregates. It has already been reported that the use of the coarse fraction of recycled masonry aggregates (MA) and recycled mixed aggregates (RMA) causes an important reduction in the abrasive resistance of the manufactured blocks [31], and, in that case, the decrease in the wear resistance was attributed to the differences in the density of the recycled aggregates. The results presented there seem to recommend the PCA of the results of the abrasion of the blocks to understand the behaviour of the manufactured elements.

The results of the independence of the slipping resistance on the properties of the recycled aggregate are in agreement with the previously published results that stated that the use of recycled aggregate has no influence on the slipping resistance of paving blocks [30,86]. Therefore, the PCA study on the slip resistance results will not be applied.

### 4.4. Leaching

With all the recycled aggregates studied, the leaching value of any metal for any type of paving block is very low, far away from the values included in the standard for the consideration of inert elements. The same result was observed by [44], but in the present paper, more types of aggregates were used and samples were produced at an industrial scale, with the relevance of that fact for the massive use of the manufactured paving blocks.

It is also noticeable that even though the sulphate content released by the aggregates MA1, MA2 and RMA was very high (up to eight times higher than the values accepted in the instruction; see Table 11), when those aggregates were used in the manufacture of paving blocks, the leaching was reduced regardless of the fraction used, coarse or fine, complying with the requirements of the NSQD regulations. So, the incorporation of recycled aggregates in the manufacture of paving blocks completely changes their leaching behaviour. This fact reveals that the manufactured paving blocks generate new products at an industrial scale that, in addition to giving value to a by-product, also decrease the risk of leaching, making more environmentally friendly products. This result is in agreement with [41], where blocks for construction were prepared using recycled aggregates, both the coarse and the fine fraction of recycled mixed aggregate formed by concrete, ceramic tiles, Arabic tiles, terrazzo, and small gypsum or wood pieces. The conclusion was reached that no significant environmental problems are expected from using the blocks with recycled aggregates.

## 5. Conclusions

In this work, the industrial manufacture of paving blocks has been studied by substituting different percentages of natural aggregates for recycled aggregates. Different types of construction and demolition waste (C&DW) as aggregates were used. The properties of these paving blocks were studied and analysed by the PCA method. Leaching behaviour has also been evaluated in the recycled aggregates and paving blocks. In accordance with this experimental study and the results obtained, the following conclusions can be reached:

- There is clear evidence of the influence of the aggregate properties on the mechanical strength of the industrially manufactured paving blocks. The properties of the aggregates should be taken into account in the different standards, or at least limiting values should be included for different properties.
- All the recycled aggregates studied, except MA2, could replace 25% of the natural aggregate in the blocks produced.
- The ceramic and mixed aggregates used in this study (MA1, MA2 and RMA) show high values for the leaching of sulphate ions, increasing the established limit in the EU directive for being considered inert according to the EU Landfill Directive. The limit for chromium is exceeded by one of the recycled ceramic aggregates (MA2). Recycled concrete aggregates are classified as inert.
- All the manufactured paving blocks in this study can be considered environmentally safe (without risk of leaching) according to the Netherland Soil Quality Decree.
- It is possible to manufacture at industrial scale paving blocks with fine and coarse RMA, CA, and MA that meet the requirements of standard UNE-EN 1338 and the Netherland Soil Quality Decree. So, the paving blocks, in addition to reusing waste, give more safe products from an environmental point of view.

## Figures and Tables

**Table 1:** Properties of natural and recycled aggregates.

Type	Recycled						Natural	
Origin	Concrete	Masonry (1)		Masonry (2)	Mixed		Natural	
Size	Coarse	Fine	Coarse	Coarse	Fine	Coarse	Fine	Coarse
Nomenclature	CA-C	MA1-S	MA1-C	MA2-C	RMA-S	RMA-C	Fine	Coarse
Water absorption (%) [51]	4.8	9.8	9.7	17.3	6.3	6.0	1.4	1.1
Dry surface density (g/cm^3^) [51]	2.39	2.15	2.15	1.85	2.32	2.32	2.67	2.70
Resistance to fragmentation [52]	28	30	30	40	30	30	24	24
Fine value (<0.063 mm) (%) [53]	1	46	2	3.1	15	1	7	1

**Table 2:** Composition of each type of recycled aggregate according to [54].

Mixture	Concrete Aggregate (CA)	Masonry Aggregate (MA1 and MA2)	Recycled Mixed Aggregates (RMA)
Floating particles	3.0%	-	6.6%
Other	0.0%	-	0.5%
Concrete	78.0%	-	22.0%
Unbound aggregate	19.0%	-	52.7%
Masonry	-	100.0%	17.2%
Asphalt	-	-	1.0%

**Table 3:** Dosages used for the manufacture of paving blocks as a function of the type of aggregate used.

Mixture	Cement (kg/m^3^)	Effective Water (l/m^3^)	Natural Aggregates 0/4 (%) ^a^	Natural Aggregates 5/12 (%) ^a^	Recycled Aggregates 0/4 (%) ^a^	Recycled Aggregates 5/12 (%) ^a^
Control	280	132	55.00	45.00	-	-
CA-C-25	280	132	55.00	33.75	-	11.25
CA-C-60	280	132	55.00	18.00	-	27.00
CA-C-100	280	132	55.00	-	-	45.00
MA1-S-25	280	132	41.25	45.00	13.75	-
MA1-S-60	280	132	22.00	45.00	33.00	-
MA1-S-100	280	132	-	45.00	55.00	-
MA1-C-25	280	132	55.00	33.75	-	11.25
MA1-C-60	280	132	55.00	18.00	-	27.00
MA1-C-100	280	132	55.00	-	-	45.00
MA2-C-25	280	132	55.00	33.75	-	11.25
MA2-C-60	280	132	55.00	18.00	-	27.00
MA2-C-100	280	132	55.00	-	-	45.00
RMA-S-25	280	132	41.25	45.00	13.75	-
RMA-S-60	280	132	22.00	45.00	33.00	-
RMA-S-100	280	132	-	45.00	55.00	-
RMA-C-25	280	132	55.00	33.75	-	11.25
RMA-C-60	280	132	55.00	18.00	-	27.00
RMA-C-100	280	132	55.00	-	-	45.00

^a^ the percentages shown are for the total aggregate in volume.

**Table 4:** Results of the eigenvalues and percentage of data variance explained by the different dimensions after the PCA applied to the samples cured for 28 and 90 days.

	Eigenvalue	Variance (%)	Cumulative Variance (%)	Eigenvalue	Variance (%)	Cumulative Variance (%)
	28 Days			90 Days		
Dim. 1	4.50	75.12	75.12	4.55	75.89	75.89
Dim. 2	1.11	18.42	93.54	1.06	17.65	93.54
Dim. 3	2.75 × 10^−1^	4.59	98.13	2.83 × 10^−1^	4.72	98.26
Dim. 4	9.48 × 10^−2^	1.58	99.71	8.87 × 10^−2^	1.48	99.74
Dim. 5	1.71 × 10^−2^	0.28	99.99	1.54 × 10^−2^	2.57 × 10^−1^	99.99
Dim. 6	1.86 × 10^−5^	3.11 × 10^−4^	100.00	1.75 × 10^−5^	2.92 × 10^−4^	100.00

**Table 5:** Weight of the different parameters for the different dimensions provided by PCA at 28 days.

	Dim. 1	Dim. 2	Dim. 3	Dim. 4	Dim. 5
Density	−0.966	0.117	−0.207	0.043	0.090
Fragmentation	0.966	−0.137	−0.025	−0.205	0.070
Floating part.	0.942	−0.246	0.005	0.224	0.046
Absorption	0.981	−0.172	0.083	0.014	−0.004
Substitution	0.353	0.908	0.224	0.023	0.026
Mech. strength	−0.815	−0.398	0.418	−0.009	0.035

**Table 6:** Result of the eigenvalues and percentage of data variance explained by the different dimensions after the PCA applied to the samples, with fine recycled aggregate, cured for 28 and 90 days.

	Eigenvalue	Variance (%)	Cumulative Variance (%)	Eigenvalue	Variance (%)	Cumulative Variance (%)
	28 Days			90 Days		
Dim. 1	4.04	67.49	67.49	4.20	70.03	70.03
Dim. 2	1.42	23.70	91.19	1.30	21.73	91.77
Dim. 3	4.94 × 10^−1^	8.24	99.43	4.61 × 10^−1^	7.68	99.45
Dim. 4	3.40 × 10^−2^	5.67 × 10^−1^	100.00	3.29 × 10^−2^	5.49 × 10^−1^	100.00
Dim. 5	5.14 × 10^−29^	8.56 × 10^−28^	100.00	5.17 × 10^−29^	8.61 × 10^−28^	100.00
Dim. 6	3.01 × 10^−31^	5.02 × 10^−30^	100.00	3.10 × 10^−31^	5.17 × 10^−30^	100.00

**Table 7:** Result of the eigenvalues and percentage of data variance explained by the different dimensions after the PCA applied to water absorption with the coarse and fine fraction at 90 days.

	Eigenvalue	Variance (%)	Cumulative Variance (%)	Eigenvalue	Variance (%)	Cumulative Variance (%)
	Coarse Fraction			Fine Fraction		
Dim. 1	4.56	75.93	75.93	4.09	68.15	68.15
Dim. 2	1.08	18.05	93.98	1.37	22.91	91.06
Dim. 3	0.26	4.28	98.26	0.53	8.78	99.84
Dim. 4	0.07	1.25	99.51	9.6 × 10^−3^	0.16	100.00
Dim. 5	0.03	0.49	100.00	5.2 × 10^−29^	8.6 × 10^−28^	100.00
Dim. 6	1.9 × 10^−5^	3.2 × 10^−4^	100.00	3.1 × 10^−31^	5.2 × 10^−30^	100.00

**Table 8:** Results of density, abrasive resistance and slipping resistance of the paving blocks after 90 days of curing.

Mixture	Density (g/cm^3^)	Abrasive Wear (mm)	Slipping Resistance
Control	2.30	16.9	86
CA-C-25	2.27	15.9	87
CA-C-60	2.24	16.3	83
CA-C-100	2.25	18.5	84
MA1-C-25	2.26	16.4	88
MA1-C-60	2.23	16.6	84
MA1-C-100	2.13	17.4	86
MA1-S-25	2.28	17.1	85
MA1-S-60	2.21	17.4	84
MA1-S-100	1.95	16.7	87
MA2-C-25	2.18	17.8	90
MA2-C-60	2.13	25.1	84
MA2-C-100	2.07	28.7	86
RMA-C-25	2.30	13.3	85
RMA-C-60	2.26	14.4	86
RMA-C-100	2.21	15.1	85
RMA-S-25	2.28	15.9	83
RMA-S-60	2.23	16.5	88
RMA-S-100	1.97	15.8	85

**Table 9:** Result of the eigenvalues and percentage of data variance explained by the different dimensions after the PCA applied to density at 90 days. Influence of the coarse aggregates.

	Eigenvalue	Variance (%)	Cumulative Variance (%)
Dim. 1	3.96	66.01	66.01
Dim. 2	1.04	17.37	83.38
Dim. 3	8.6 × 10^−1^	16.34	97.72
Dim. 4	9.5 × 10^−2^	1.58	99.30
Dim. 5	4.2 × 10^−2^	7.0 × 10^−1^	99.99
Dim. 6	1.9 × 10^−5^	3.2 × 10^−4^	100.00

**Table 10:** Result of the eigenvalues and percentage of data variance explained by the different dimensions after the PCA applied to abrasive resistance at 90 days. Influence of the coarse aggregates.

	Eigenvalue	Variance (%)	Cumulative Variance (%)
Dim. 1	4.47	74.57	74.57
Dim. 2	0.99	16.54	91.11
Dim. 3	4.21 × 10^−1^	7.01	98.12
Dim. 4	9.07 × 10^−2^	1.51	99.63
Dim. 5	2.17 × 10^−2^	3.6 × 10^−1^	100.00
Dim. 6	1.63 × 10^−5^	2.7 × 10^−4^	100.00

**Table 11:** Concentrations of metals, chloride and sulphate in leachate and classification according to the EU Landfill Directive applied to recycled aggregates.

Limit Imposed EU Landfill Directive L/S = 10 L/kg (mg/kg)		Concrete Aggregates	Masonry Aggregates			Recycled Mixed Aggregates	
		CA-C	MA1-S	MA1-C	MA2-C	RMA-S	RMA-C
Cr	0.5	0.140	0.117	0.105	0.513	0.111	0.154
Ni	0.4	0.014	0.009	0.009	0.040	0.017	0.020
Cu	2.0	0.014	0.009	0.009	0.117	0.077	0.017
Zn	4.0	0.043	0.000	0.000	0.006	0.000	0.000
As	0.5	0.009	0.026	0.020	0.006	0.009	0.034
Se	0.1	0.011	0.006	0.006	0.040	0.060	0.000
Mo	0.5	0.057	0.074	0.063	0.123	0.009	0.043
Cd	0.0	0.000	0.000	0.000	0.003	0.000	0.000
Sb	0.1	0.006	0.009	0.003	0.006	0.000	0.006
Ba	20.0	0.889	0.573	0.590	1.342	0.875	0.633
Chloride	800.0	796.842	581.229	529.519	805.410	1743.148	720.124
Sulphate	1000.0	26.391	4928.790	4927.080	3722.100	8195.460	4910.550
Fluoride	10.0	1.107	1.032	0.990	1.095	1.390	0.955
Classification according to concentration on heavy metals and sulphates (EU landfill Directive)		Inert	Non-hazardous	Non-hazardous	Non-hazardous	Non-hazardous	Non-hazardous

**Table 12:** Concentrations of metals, chloride and sulphate in leachate and limits of the Netherland Soil Quality Decree applied to the paving blocks.

Element	NSQD Limits (mg/m^2^)	CONTROL (mg/m^2^)	CA-C-100 (mg/m^2^)	MA1-S-100 (mg/m^2^)	MA1-C-100 (mg/m^2^)	MA2-C-100 (mg/m^2^)	RMA-S-100 (mg/m^2^)	RMA-C-100 (mg/m^2^)
Cr	120	0.650	0.217	1.408	0.650	4.442	0.650	0.433
Ni	81	0.000	0.000	0.000	0.000	0.000	0.000	0.000
Cu	98	0.000	0.000	0.000	0.000	0.000	0.000	0.000
Zn	800	0.000	0.000	0.000	0.000	0.000	0.000	0.000
As	260	0.108	0.108	0.108	0.108	0.000	0.108	0.108
Se	4.8	0.000	0.000	0.000	0.000	0.000	0.000	0.000
Mo	144	0.758	0.000	0.000	0.000	0.000	0.000	0.000
Cd	3.8	0.000	0.000	0.000	0.000	0.000	0.000	0.000
Sb	8.7	0.000	0.000	0.000	0.000	0.000	0.000	0.000
Ba	1500	0.758	3.358	2.708	2.383	6.175	2.708	2.708
Chloride	110,000	349.917	1258.292	561.167	999.483	881.833	228.583	321.642
Sulphate	165,000	88.833	1001.758	7480.850	2703.350	5812.517	1284.942	1222.758
Fluoride	2500	14.733	17.008	16.467	13.542	17.442	1.300	1.620

## Data Availability

The original contributions presented in the study are included in the article/Supplementary Materials, further inquiries can be directed to the corresponding author.

## Supplementary Materials

The following supporting information can be downloaded at: https://www.mdpi.com/article/10.3390/ma17122898/s1.
