# Supplementary material for: Influence of the Properties of Different Types of Recycled Aggregate on the Service Properties and Leaching of Paving Blocks Manufactured at Industrial Scale
Source: Materials (Basel). 2024 Jun 13;17(12):2898. doi: 10.3390/ma17122898 (PMC11205776; doi:10.3390/ma17122898)
Supplement: Supplementary file 1 [file materials-17-02898-s001.zip › materials-3047404-supplementary.pdf]

In order to prove the relevance from the statistical point of view of the data used for the PCA analysis, authors compare for two different situations the results of the PC analysis for the experimental data used, and a new dataset, formed by the experimental data and 30 more random results that fulfill the average value and the standard deviation of the experimental data. The calculation has been done for the mechanical strenght of samples with substitution of coarse aggregate at 28 days and for the water absorption of the blocks where the fine fraction of the recycled aggregate has been used.

<u>Mechanical strength of paving blocks with recycled coarse aggregate at 28 days</u>, with two data sets. The first one uses the 8 expeimental results, and the second one adds 30 random simulated results that fulfill the average value for the mechanical strength.

Comparison among the eigenvalues, variance and cumulative variance obtaind with experimental data, 8 samples (blue) and experimental +simulated data 38 samples (green).

	Eigenvalue	Variance (%)	Cumulative variance (%)	Eigenvalue	Variance (%)	Cumulative variance (%)
	Experimental data 28 days			Experimental+simulated data, 28 days		
Dim. 1	4.50	75.12	75.12	4.5	75.07	75.07
Dim. 2	1.11	18.42	93.54	1.11	18.42	93.49
Dim. 3	2.75e-01	4.59	98.13	2.78e-1	4.64	98.13
Dim. 4	9.48e-02	1.58	99.71	9.48e-2	1.58	99.71
Dim. 5	1.71e-02	0.28	99.99	1.75e-2	0.29	99.99
Dim. 6	1.86e-05	3.11e-04	100.00	1.87e-5	3.01-04	100.00

Comparison of the weights for the different aggregate properties in each dimension.

	Experimental data (8 samples)					Exp.+simulated data (38 samples)				
	Dim. 1	Dim. 2	Dim. 3	Dim. 4	Dim. 5	Dim. 1	Dim. 2	Dim. 3	Dim. 4	Dim. 5
Density	-0.966	0.117	-0.207	0.043	0.090	-0.966	0.116	-0.206	0.043	0.092
Fragmentation	0.966	-0.137	-0.025	-0.205	0.070	0.966	-0.137	-0.024	-0.205	0.071
Floating part.	0.942	-0.246	0.005	0.224	0.046	0.942	-0.245	0.006	0.223	0.046
Absorption	0.981	-0.172	0.083	0.014	-0.004	0.981	-0.172	0.083	0.014	-0.004
Substitution	0.353	0.908	0.224	0.023	0.026	0.353	0.907	0.226	0.023	0.026
Mech. strength	-0.815	-0.398	0.418	-0.009	0.035	-0.813	-0.399	0.421	-0.009	0.035

Comparison of the correlation circles

Experimental data.

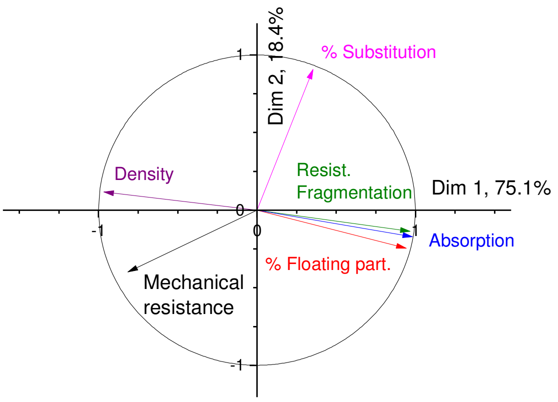

Experimental+simulated data

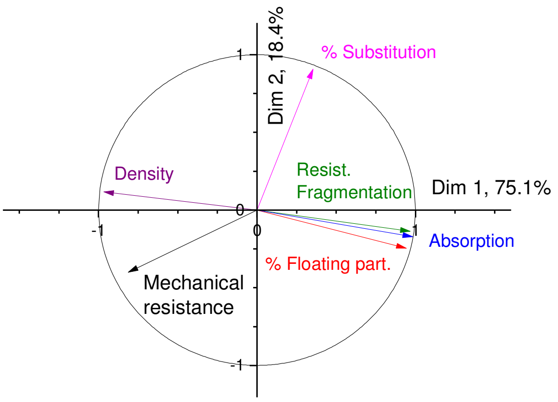

<u>Water absorption fine recycled aggregates at 90 days</u> with two data sets. The first one uses the 8 experimental results, and the second one adds 30 random simulated results that fulfill the average value for the water absorption.

	Eigenvalue	Variance (%)	Cumulative variance (%)	Eigenvalue	Variance (%)	Cumulative variance (%)
	Experimental data			Experimental+simulated data		
Dim. 1	4.09	68.15	68.15	4.09	68.15	68.15
Dim. 2	1.37	22.91	91.06	1.37	22.91	91.06
Dim. 3	0.53	8.78	99.84	0.53	8.77	99.83
Dim. 4	9.6e-3	0.16	100.00	9.7e-3	0.16	100
Dim. 5	5.2e-29	8.6e-28	100.00	4.3e-28	7.1e-27	100
Dim. 6	3.1e-31	5.2e-30	100.00	2.4e-29	4.0e-28	100

Comparison of the weights for the different aggregate properties in each dimension.

	Experimental data (8 samples)					Exp.+simulated data (38 samples)				
	Dim. 1	Dim. 2	Dim. 3	Dim. 4	Dim. 5	Dim. 1	Dim. 2	Dim. 3	Dim. 4	Dim. 5
Density	-0.961	0.268	0.069	0.007	0.000	-0.961	0.268	0.069	0.007	0.000
Fragmentation	0.860	-0.103	-0.499	-0.024	0.000	0.860	-0.103	-0.499	-0.024	0.000
Floating part.	0.800	-0.385	0.460	0.015	0.000	0.800	-0.385	0.460	0.015	0.000
Absorption	0.951	-0.304	0.057	-0.002	0.000	0.951	-0.304	0.057	-0.002	0.000
Substitution	0.740	0.664	-0.078	0.072	0.000	0.740	0.664	-0.078	0.072	0.000
Block Absorption	0.579	0.781	0.226	-0.060	0.000	0.579	0.781	0.226	-0.061	0.000

The correlation circles have not been included in this comparison, because seing the weights of each propertie on dimension 1 and 2 readers can notice that the circles will be imposible to distinguish.

Conclusion

As it can be seen with this comparison the number of samples included in the paper, following the standard, are representative for the principal component analysis and including a bigger number of samples will not change the statistical results.
